# Exercise to Reduce Anthracycline-Mediated Cardiovascular Complications in Breast Cancer Survivors

**DOI:** 10.3390/curroncol28050351

**Published:** 2021-10-13

**Authors:** Sonu S. Varghese, Will J. Johnston, Cameron R. Eekhoudt, Melanie R. Keats, Davinder S. Jassal, Scott A. Grandy

**Affiliations:** 1 Institute of Cardiovascular Sciences, St. Boniface Hospital Albrechtsen Research Centre, University of Manitoba, Winnipeg, MB R2H 2A6, Canada; varghese1@myumanitoba.ca (S.S.V.); eekhoudc@myumanitoba.ca (C.R.E.); djassal@sbgh.mb.ca (D.S.J.); 2 School of Health & Human Performance, Faculty of Health, Dalhousie University, 6230 South Street, Halifax, NS B3H 4R2, Canada; WillJohnston@dal.ca (W.J.J.); melanie.keats@dal.ca (M.R.K.); 3 Beatrice Hunter Cancer Research Institute, Halifax, NS B3H 4R2, Canada; 4 Department of Medicine, Nova Scotia Health, Central Zone, Halifax, NS B3H 1V7, Canada; 5 Section of Cardiology, Department of Internal Medicine, Max Rady College of Medicine, Rady Faculty of Health Sciences, University of Manitoba, Winnipeg, MB R3E 0W2, Canada

**Keywords:** exercise, breast cancer, anthracycline, cardiotoxicity, cardioprotection, aerobic, resistance

## Abstract

While developments in cancer therapeutics have greatly reduced morbidity and mortality in females with breast cancer, it comes at a cost of an increased risk of cardiovascular complications. In particular, anthracyclines, like doxorubicin, which are a mainstay of current chemotherapy regimens, are associated with dose-dependent cardiotoxicity. Exercise has been widely accepted as an effective intervention in reducing cardiovascular risk in a variety of different clinical conditions. However, the benefits of exercise in anthracycline-mediated cardiotoxicity are not clearly understood. First, this review discusses the pre-clinical studies which have elucidated the cardioprotective mechanisms of aerobic and resistance exercise in improving cardiovascular function in the setting of anthracycline treatment. Next, it aims to summarize the results of aerobic and resistance exercise clinical trials conducted in females with breast cancer who received anthracycline-based chemotherapy. The review further discusses the current exercise guidelines for women undergoing chemotherapy and contraindications for exercise. Finally, the review addresses gaps in research, specifically the need for further clinical trials to establish a recommended exercise prescription within this patient population.

## 1. Introduction

Cancer and heart disease remain the leading causes of death worldwide [1]. Despite tremendous improvements in cancer therapeutics, collateral damage to the heart remains the leading cause of morbidity and mortality among breast cancer (BC) survivors [2]. Depending on the stage and molecular features of the cancer cells, treatment may include surgery, radiation therapy, chemotherapy, and/or targeted therapies [3]. While endorsing a multimodal treatment plan is fundamental for improving the longevity of breast cancer survivors, many of these treatment modalities are associated with detrimental cardiotoxicity [2]. Among breast cancer survivors, there is an increased risk of several cardiovascular side effects including: (i) cardiomyopathy; (ii) arrhythmia; (iii) valvular disease; and/or (iv) heart failure [4,5,6,7,8]. Additionally, age, adjuvant chemotherapy, and traditional cardiac risk factors such as a prior cardiac event, family history of coronary artery disease (CAD), smoking, and hypertension increase the likelihood of developing cardiotoxicity from anti-cancer therapy [9,10]. For the purposes of this review, breast cancer survivorship begins at diagnosis and continues during treatment and through the rest of a person’s life [11]. 

Due to its potent, broad-spectrum antineoplastic properties, anthracycline-based chemotherapy remains the leading class of cytotoxic agents used in the setting of breast cancer [2]. The anthracycline class of chemotherapy contains several effective agents including epirubicin, idarubicin, and most commonly doxorubicin (DOX) [2]. While the use of DOX has facilitated a reduction in cancer relapse and mortality in the breast cancer setting, its clinical utility is limited by dose-dependent cardiotoxicity [2,12]. While the clinical use of anthracyclines has decreased due to the emergence of novel agents with improved therapeutic indexes, their use remains mainstay in the treatment of a variety of indications. As such, treatment protocols associated with anthracycline-based chemotherapeutic regimens have changed to reduce the risk of cardiotoxicity. Specifically, reducing the rate of administration and altering the formulation of anthracyclines have proven successful at reducing the associated cardiotoxic risk [13]. It is widely accepted that DOX induces cardiotoxicity through several pathways, including DNA topoisomerase II inhibition, oxidative damage and free radical generation, and cardiomyocyte apoptosis [14]. Importantly, while stringent recommendations including a maximum cumulative dose of 500 mg/m^2^ of DOX has been established to mitigate potential cardiotoxic risk, there is no accepted dose that is considered to be without risk [4,15]. Furthermore, the addition of targeted therapies including trastuzumab (TRZ) in the treatment of HER2+ breast cancer further compounds the risk of cardiotoxicity with incidences occurring in up to 1 in 4 individuals [9,16,17]. Importantly, while adjuvant TRZ therapy substantially exacerbates anthracycline-mediated cardiotoxicity, studies have shown that TRZ monotherapy is associated with up to 7% risk of cardiotoxic side effects [18].

## 2. Detecting Cardiotoxicity

The American Society of Echocardiography and the European Association of Cardiovascular Imaging established a consensus to define cancer therapeutics-related cardiac dysfunction (CTRCD) as a decrease in left ventricular ejection fraction (LVEF) of >10 percentage points, to a value <53% as determined by two-dimensional echocardiography [19]. Moreover, the echocardiographic study should be repeated 2 to 3 weeks after baseline examination to corroborate initial findings [19]. Additionally, the degree of cardiac dysfunction can be further classified as reversible, partially reversible, and irreversible depending on the degree of LVEF recovery from the nadir [19]. There has been an increased focus on the early detection of symptomatic and asymptomatic CTRCD to avoid both cancer treatment delays and significant cardiovascular damage [20]. Determining indices of cardiotoxicity prior to the onset of overt CTRCD is important to prevent irreversible cardiac damage. Specifically, the novel use of global longitudinal strain (GLS) has been shown to be a sensitive indicator of early LV systolic dysfunction among patients with CTRCD [21]. In addition to detecting early myocardial changes, GLS has been shown to provide valuable insight regarding reversibility of LV systolic dysfunction among patients receiving cancer therapy [21].

## 3. Prevention of Anthracycline-Mediated Cardiotoxicity

In addition to discovering reliable and sensitive methods to detect CTRCD, determining novel primary preventative strategies for CTRCD continues to galvanize cardio-oncology research. Currently, dexrazoxane, an iron-chelating agent, remains the only FDA approved cardioprotective agent used to reduce the risk of cardiac dysfunction resulting from cardiotoxic chemotherapy [22]. While both pre-clinical and clinical research has investigated the cardioprotective effects of pharmaceuticals including β-blockers, renin-angiotensin-aldosterone system antagonists, and statins, their use in preventing chemotherapy-induced cardiotoxicity remains limited in the clinical setting [19]. Additionally, undesirable side effects of these medications can include reduced blood pressure leading to worsening fatigue and light-headedness among cancer patients.

Physical activity is a widely endorsed intervention by both cardiologists and oncologists for its variety of physiological benefits including reduced risk of cardiovascular events, weight management, and improved quality of life among patients [23,24]. Early evidence has shown that physical exercise during cancer therapy is a safe and feasible intervention that may improve physical functioning, fatigue, and quality of life measures [25]. As a result, the American Heart Association recommends that high risk cardio-oncology patients follow an individualized rehabilitation program [26]. Additionally, the American College of Sports Medicine recommends cancer patients perform 75 min of vigorous aerobic physical activity or 150 min of moderate intensity aerobic physical activity or an equivalent combination per week [27]. This was updated in 2019 by the Consensus Statement from the International Multidisciplinary Roundtable on Exercise Guidelines for Cancer Survivors which recommended at least 90 min of moderate-intensity exercise per week in cancer survivors [27]. Despite the corroborating evidence supporting the potential physiological benefits of physical activity during cancer therapy, its role in preventing CTRCD remains limited. Herein, we summarize the current basic science and clinical research surrounding the potential effects of physical exercise in reducing the risk of chemotherapy-induced cardiotoxicity among breast cancer survivors.

## 4. Exercise: Aerobic and Resistance

Clinically, aerobic exercise (AE) can be defined as structured, prolonged activity which involves a sustained increase in heart rate resulting in heightened oxygen consumption and utilization. The goal of AE is to achieve an increase in aerobic capacity and improve cardio-pulmonary health. Aerobic capacity can be thought of as the body’s dual ability to supply oxygen to and remove by-products from the various tissues of the body. Aerobic capacity is best assessed using a measure of peak oxygen consumption and is expressed in units of L/min (e.g., absolute VO_2_) or mL/kg/min (e.g., relative VO_2_). In pre-clinical studies, AE has been simulated in animals through motorized treadmills, running wheels, and swimming exercises. 

While AE depends more heavily on oxygen-dependent pathways involved in cellular respiration, resistance training (RT) primarily utilizes ATP without the need for an increased oxygen consumption [28,29]. Specifically, RT utilizes the phospho-creatine and glycolysis energy pathways to meet the necessary energy demands of RT exercise. In clinical studies, RT often consist of completing 1–3 sets of an exercise with each set consisting of 8–12 repetitions. These exercises target different large muscle groups and an increase in difficulty is achieved by increasing load, repetitions, and/or sets. In pre-clinical studies, RT has been simulated by encouraging animals to lift loads by standing upright in a bipedal stance.

## 5. Basic Science: Aerobic and Resistance Exercises

There have been several pre-clinical exercise studies, both AE and RT, which have investigated the benefits of exercise in models of chemotherapy induced cardiotoxicity. Both AE and RT have shown its cardioprotective benefits, which are primarily due to their ability to attenuate DOX-induced increases in oxidative stress and apoptosis (Table 1 and Table 2).

An acute exercise study by Wonders et al. showed a single bout of treadmill running 24 h prior to DOX injection in rats resulted in lower levels of myocardial lipid peroxidation, a marker of oxidative stress [30]. This was also corroborated by chronic exercise studies which found attenuated levels of myocardial lipid peroxidation in rats that ran on treadmills or wheels prior to DOX treatment compared to their sedentary counterparts [31,32,33]. Most recently, Phungphong et al. showed a 10-week exercise program led to the attenuation of DOX-induced increases in serum protein carbonylation, a marker of oxidative stress [34]. 

These exercise-induced reductions in oxidative stress are thought to be in part due to an increase in antioxidant expression. To this end, Ascensao et al. showed endurance swim training for 14 weeks led to increases in antioxidant glutathione expression in hearts of DOX-treated mice [35]. Further, the same research team later investigated the benefits of a 14-week motorized treadmill running program in DOX-treated mice [36]. They found that exercise training was able to increase the antioxidant enzyme superoxide dismutase (SOD) [36]. Exercise-induced increases in SOD have also been corroborated by other pre-clinical studies [32,33,37]. These studies have also shown increases in other antioxidant enzymes with exercise training in the setting of DOX-treated animals, including catalase and glutathione peroxidase [37].

Finally, Ascensao et al. investigated the benefits of a 14-week motorized treadmill running program in DOX-treated mice [36]. They found that exercise training was able to attenuate DOX-induced increases in pro-apoptotic proteins including Bax and cleaved caspase 3 [36]. The ability of AE to attenuate cleaved caspase 3 activity has also been observed in other chronic exercise studies [34,38]. The reductions in these pro-apoptotic markers indicate exercise can decrease cardiomyocyte apoptosis in the setting of DOX leading to preserved heart structure and function. Important to note, these studies showed similar results in reducing pro-apoptotic markers despite the length of the training program, which varied from as short as 2 weeks to as long as 14 weeks [34,36,38].

While several pre-clinical studies have investigated the role of AE in the setting of DOX-induced cardiotoxicity, studies exploring the benefits of RT are more limited (Table 2). It has been previously shown that DOX induces a change in the muscle fiber composition of the heart [39]. Particularly, DOX induces a change in myosin heavy chains (MHC) isoform distribution by upregulating beta-MHC and downregulating alpha-MHC. Typically, in a healthy rat heart, the alpha-MHC is the major isoform, and it is associated with increased ATPase activity and contractility. As such, this DOX-induced shift in isoforms leads to weaker cardiac contractility and decreased cardiac force production. A study by Pfannenstiel et al. showed rats that performed a 12-week resistance training period prior to DOX administration were able to prevent cardiomyocyte MHC isoform shifts [40]. The resistance training program consisted of progressively increasing the elevation of food and drink which necessitated the rise to a bipedal stance for the animals to eat and drink. In addition to maintaining MHC isoform distribution, the trained rats also had reduced septal wall thinning, preserved mass of left ventricle and reduced cardiac dysfunction. Therefore, this study indicated that RT may provide cardioprotective benefits in the setting of DOX-induced cardiotoxicity by preventing a MHC isoform shift. Most recently, a study by Feitosa et al. showed how an 8-week RT program in DOX-treated rats was able to attenuate increases in diastolic arterial pressure, heart rate, sympathetic tone, and oxidative stress [41].

**Table 1:** Pre-clinical Aerobic Exercise Studies.

Animal Species	Doxorubicin Administration (Drug/Dose/Route)	Aerobic Exercise Regimen (Type/Duration/Max Speed/Max Grade)	Major Findings	Reference
Female Sprague Dawley rats	2.5 mg/kg given every other day (i.p), 6 injections in total	Motor-driven treadmill; 21 m/min; started at 0% grade for 20 min and progressed to 5.5% grade for 60 min; 5 days/week; 10 weeks	- Attenuation of doxorubicin-induced increases in cleaved caspase 3 - Attenuation of serum protein carbonylation - Partial attenuation of IL-6 elevations	Phungphong et al. (2020) [34]
Male Sprague Dawley rats	15 mg/kg DOX single-dose (i.p.)	Motorized treadmill; 60 min; 25 m/min; 5% grade	Exercise preserved end-systolic pressure in doxorubicin-treated rats - lipid peroxidation levels were compared to control group	Wonders et al. (2008) [30]
Male Charles River Cd1 mice	20 mg/kg DOX single-dose (i.p.)	Swimming; 1 h/day, 5 days/week for 14 weeks.	- Training led to increased total and reduced glutathione - Attenuated increase in HSP60 expression, marker of acute cell stress	Ascensão et al. (2005) [35]
Male Wistar rats	20 mg/kg DOX single-dose (i.p.)	Motor-driven treadmill; 30 m/min; 0% grade for 60 min/day; 5 days/week for 14 weeks	- Training increased antioxidant gene expression (Mn-SOD, Cu-Zn SOD) - Training restored DOX-induced increases in proapoptotic proteins Bax and cleaved-caspase 3	Ascensão et al. (2005) [36]
Male Sprague Dawley rats	2.5 mg/kg DOX given 3 days/week (i.p.)—cumulative dosage of 15 mg/kg	Motorized treadmill; 15 mL/min, 20 min/day, 5 days/week for 2 weeks	- Low intensity exercise led to attenuation of DOX-induced increases in cleaved caspase-3 activity	Chicco et al. (2006) [38]
Female Sprague Dawley rats	Isolated hearts were perfused with Krebs–Henseleit buffer containing 10-uM DOX	Voluntary running with access to wheel 24 h/day for 8 weeks	- Attenuated levels of myocardial lipid peroxidation	Chicco et al. (2005) [35]
Male Sprague Dawley rats	20 mg/kg single-dose DOX (i.p.)	Treadmill; Maximum of 60 min/day at 30 m/min, 0% grade for 10 days.	- Exercise increased levels of SOD1, SOD2, GPX1, and catalase	Kavazis et al. (2010) [37]
Male Wistar rats	20 mg/kg DOX single-dose	Treadmill; 15–17 m/min for 25–39 min/day for 5 days/week for 3 weeks; 0% grade	- Exercise decreased levels of MDA - Increased levels of SOD	Ahmadian et al. (2017) [33]

Dox = doxorubicin; i.p = intraperitoneal injection; IL = interleukin; HSP = heat shock protein; SOD = superoxide dismutase; GPX = glutathione peroxidase; MDA = malondialdehyde.

**Table 2:** Pre-clinical Resistance exercise studies.

Animal Species	Doxorubicin Administration (Drug/Dose/Route)	Resistance Exercise Type	Major Findings	Reference
Male Sprague-Dawley rats	12.5 of DOX mg/kg single-dose bolus (i.p.)	Animals placed in a cage that provided food/water at an elevated height which encouraged rising to a bipedal stance to eat and drink. Height was increased from standard of 20.32 cm to a maximum of 35.5 cm. Training was a total of 12 weeks.	- reduced cardiac beta myosin heavy chain isoform distribution	Pfannenstiel et al. (2018) [40]
Male Wistar rats	2.5 mg/kg DOX administered per week for 6 weeks (i.p.)	Animals were placed in an upright position, standing on lower limbs and a weighted exercise apparatus attached to a vest worn by animals. Resistance program consisted of 3 sets of 10 repetitions, lifting 40% of 1RM. Rest period of 60 s between sets, repeated 3 times a week for 8 weeks. One repetition maximum test was conducted by calculating the maximum weight animals could lift. Electrical stimulus was applied to tail through electrode to initiate lifting action.	- training program in DOX-treated rats were able to attenuate increases in diastolic arterial pressure, heart rate, sympathetic tone and oxidative stress	Feitosa et al. (2021) [41]

Dox = doxorubicin; i.p. = intraperitoneal injection; 1 RM = one-repetition maximum.

Taken together, these pre-clinical studies show both aerobic and resistance exercises provide cardio-protection through the reduction in DOX-induced cardiotoxicity. As many of the studies vary in duration of exercise sessions, length of training program, type of exercise, and animal model, they are able to demonstrate the many different cardioprotective mechanisms of exercise. While basic science studies are able to elucidate the mechanisms, clinical trial studies are required to understand the feasibility and clinically relevant benefits in women with breast cancer.

## 6. Effects of Aerobic Exercise on CTRCD

A strong relationship between cardiorespiratory fitness and the risk of cardiovascular events and mortality exists in a variety of clinical patients including BC survivors after chemotherapy treatment [42]. In fact, BC survivors are at a higher risk of cardiovascular disease mortality compared to BC related mortality [43,44]. Cardiorespiratory fitness is adversely impacted during administration of chemotherapy, affecting respiratory, cardiovascular, and skeletal muscle functions [43,45]. Poor cardiorespiratory fitness also has direct consequences on the performance of activities of daily living, and thus impacts patients’ quality of life [46,47]. A study consisting of 222 BC patients, published by Klassen et al. found that BC patients have significantly impaired cardiopulmonary function (VO_2peak_) during and after chemotherapy (*p* < 0.001) [43]. The authors note that chemotherapy appears to impair cardiorespiratory fitness by influencing the oxygen delivery system rather than impacting metabolic muscle function [43]. Similarly, Giallauria et al. published a study to investigate whether exercise training improves cardiopulmonary and endothelial function in women with BC (Table 3) [44]. A total of 51 females with a history of BC were recruited for the study and asked to cycle on a stationary bike 3 times per week at 60–70% VO_2peak_ for 12 months. Cardiopulmonary and endothelial function were assessed at baseline and at 1-year follow up. Overall, they found that exercise training prevents further reductions or improves cardiopulmonary and endothelial functions among BC patients [42,44]. More recently, a 2020 randomized controlled trial (RCT) conducted by Scott and colleagues investigated the effects of two separate exercise prescription schedules and their respective influence on cardiorespiratory function in females with post-treatment for primary BC. This RCT randomly allocated 174 females to either 1 of 2 supervised exercise training interventions delivered with a fixed dose (i.e., fixed dose of 160 min/week walking), or variable dose (i.e., varied dose averaging 120 min/week walking) for 16-weeks. The primary proxy measure for cardiorespiratory fitness was VO_2peak_ measured at baseline and post-intervention. Study findings show that short term exercise training, independent of dosing schedule, is associated with improvements in cardiorespiratory fitness in patients previously treated for early stage breast cancer [48]. Taken together, the aforementioned evidence supports that cardiorespiratory fitness is lost following administration of DOX chemotherapy and that AE training can help attenuate losses seen in VO_2peak_, and thus decrease the risk of cardiac dysfunction and cardiovascular mortality [43,46].

To address the notion that exercise training should be initiated prior to, or shortly after chemotherapy treatment, Kirkham et al. investigated the acute cardiovascular changes that occur in BC survivors who randomized to exercise prior to DOX chemotherapy (i.e., 24 h prior to a single dose and 24 h prior to each and every dose of DOX) [49,50]. Results showed that the first DOX treatment is associated with a transient increase in N-terminal pro b-type natriuretic peptide (NT-proBNP) levels, undesirable echocardiographic parameters of myocardial relaxation, LV volume overload, and changes in longitudinal strain, all of which are indicative of cardiotoxicity [49,50]. However, exercise sessions performed 24 h prior to a single dose of treatment attenuated the NT-proBNP release and improved systolic function but did not have an effect on subclinical markers (i.e., 10% reduction in LVEF and GLS, presence of cardiac biomarkers (i.e., troponins)) of cardiotoxicity on repeated doses over a course of treatment [49,50]. Although future investigations are needed to corroborate these findings in a larger randomized control trial, it offers convincing evidence supporting the use of AE in BC populations. In parallel with the emerging evidence supporting preventative prescription of AE among BC patients, Keats et al. published a feasibility study outlining a 12-week, progressive, AE program that ranged from low-vigorous intensity and was administered during and after chemotherapy treatment [51]. This pilot study demonstrated that an individualized AE plan is a safe, effective and feasible program and can be effectively used to explore the effects of exercise on chemotherapy-induced cardiotoxicity [51].

While the majority of studies demonstrate that AE is a viable strategy to improve cardiorespiratory fitness in BC patients receiving chemotherapy, [41,43,48,49,50] these trials focused primarily on continuous low-moderate intensity AE, performed at different frequencies and durations. As a result, these studies did not assess the potential cardioprotective effects of higher intensity exercise. This makes it difficult to establish a consensus regarding the optimal type, duration, and intensity of exercise interventions among these patients. To further characterize the optimal type, timing, and intensity of AE, Lee et al. initiated a pilot study with the purpose of determining whether higher-intensity training elicits favorable cardiac adaptations in BC survivors [52]. To achieve this, the researchers utilized an 8-week high intensity interval training (HIIT) program to test the feasibility of the proposed exercise strategy for women with BC. The researchers hypothesized that HIIT could induce preferred cardiovascular adaptions in BC patients. While the study did not have sufficient power to determine the program’s overall effectiveness on health outcomes, they found that HIIT for BC patient’s receiving chemotherapy is feasible with a session attendance rate of 82.3% and a retention rate of 100% [52]. Lee et al. reported no significant differences in the observed adherence and retention rates compared to that of moderate intensity continuous exercise [52]. Following this pilot project, a study conducted by Uth et al. examined four different exercise intensities as defined by max heart rate (HRmax) (60–70%, 70–80%, 80–90%, and >90% HRmax) via the use of sports in a cohort of BC patients. Of note, the exercise intensity interval with the most amount of time spent in by participants was the high intensity zone (>90% HRmax). Contradictory to prior studies, this research did not demonstrate significant outcomes regarding markers of cardiac dysfunction, including cardiorespiratory fitness, resting heart rate and blood pressure, and body fat mass. However, as mentioned by the study’s limitations, the exercise opportunities were only offered a maximum of 2 days per week which may have prevented the accrual of cardioprotective benefits [53]. From these investigations, it is clear that additional studies with more rigorous scientific methodology are needed to corroborate the cardioprotective capabilities of AE in BC patients. 

## 7. Combinational Effects of Aerobic Exercise and Resistance Training on CTRCD 

It is well-established that RT is safe and beneficial for attenuating many oncological treatment-related side-effects including fatigue, lower physical functioning and diminished quality of life parameters [54]. This has led to recommendations put forth by international agencies supporting the idea that exercise, including RT, should be implemented in support of care across the cancer-care continuum [54]. Nevertheless, despite considerable progress in research and growing recognition regarding the benefits of exercise in cancer patients, current recommendations regarding RT exercise remain relatively basic and underdeveloped [54,55]. Current resistance training guidelines for exercise-oncology applications are similar to standard guidelines, at least 2 sessions per week of large muscle group strength training, doing at least 2 sets of 8 to 15 repetitions, using a weight or resistance that is at least 60% of a person’s one-repetition maximum [27,56]. This poses a major problem in the effective utilization of exercise training given the tremendous heterogeneity of cancer patients [55]. Given the multitude of RT programs that can be utilized to obtain specific training adaptations, it has been proposed that broadly applying a generic prescription to such a diverse population may limit the full therapeutic potential of RT in the cancer rehabilitation setting [54]. Therefore, guidelines with a greater degree of specificity to this target population is an important avenue of scientific and clinical inquiry. Within the realm of breast cancer and the associated treatment induced cardiac damage, consistent with claims outlined previously, the focus in the literature is primarily on AE, with little research investigating RT exclusively. In fact, the primary prescription of RT within the literature, to date, is exclusively coupled with AE. Moreover, it is hypothesized that an exercise program consisting of a combined prescription of both AE and RT may induce favorable physiological outcomes when compared to either modality prescribed independently [57,58,59]. To evaluate the application of combination training in BC populations, a randomized controlled trial by Cornette et al. sought to investigate the effects of a two-arm prospective home-based exercise program consisting of both aerobic and RT prescribed to a BC population (Table 4). Outcome measures of the study included aerobic capacity, muscular strength as well as patient fatigue. For the purposes of this review, only indices related to cardiac function will be discussed. The study recruited 44 BC patients receiving adjuvant or neoadjuvant chemotherapy. Participants were randomly assigned to either exercise intervention or control groups in a 1:1 ratio. The exercise intervention consisted of a home-based combination training program (endurance and resistance) where participants participated in AE for a minimum of 3 times per week for a total of 27 weeks. Each aerobic training session consisted of a 5-min warm-up, 20–40 min AE (i.e., walking/stationary bike) and a 5-min cool-down. Strength/RT was performed once per week and consisted of exercises targeting 5 core muscle groups (i.e., abdominal, hamstring, quadriceps, triceps and gluteus maximus). After 27-weeks of combination training, aerobic capacity increased significantly in the exercise group compared to the control group; however, no significance was detected in an intent-to-treat analysis (i.e., all participants randomized are included in the statistical analysis and analyzed according to the group they were originally assigned, regardless of what treatment (if any) they received). Furthermore, at 54-week follow-up, significance was not maintained between exercising and control groups, although significant increases in VO_2peak_ were maintained within group 54-weeks post training. This suggests that strategies to keep patients interested in participation in exercise training post-intervention are needed [60]. 

Following the work by Cornette et al., an investigation conducted by Mijwel et al. looked to contrast two combination interventions, each consisting of two supervised sessions per week. Participants were randomized to control, or either concurrent high intensity interval AE and RT (RT-HIIT) or concurrent moderate-intensity continuous AE and RT (AT-HIIT) [58]. Two hundred and forty women were randomized to 16 weeks of RT-HIIT, AT-HIIT, or control. Outcome measures of this investigation included cardiorespiratory fitness, muscle strength, body mass, hemoglobin levels, and pressure-pain threshold [58]. Overall, results from the investigation highlight that sixteen weeks of RT-HIIT was superior to AT-HITT in improving muscle strength and reduced pain sensitivity [58]. There were no differences found between exercise programs in regard to exercise tolerance and efficiency in preventing increases in body mass and increasing cardiorespiratory fitness [58]. These results corroborate that implementing a combination of resistance and high-intensity interval training during chemotherapy is importance for women with BC. 

Due to the favorable adaptations seen in combination training, particularly when RT and high intensity AE are used in tandem, new therapeutic exercise trials are warranted to elucidate best prescription practices for exercise training. This will support improved cardiovascular indices both during and following cancer therapy. Specifically, to expand on the evaluation of utilizing a combination of high-intensity aerobic training and RT in exercise prescription, a study conducted by An et al. sought to provide early randomized comparisons of different doses and types of exercise performed during breast cancer chemotherapy on longer-term patient-reported outcomes, health-related fitness, and exercise behavior [59]. Specific results from this investigation report show that combination training was significantly superior to only high-intensity aerobic training on upper body muscular endurance and on lower body muscular strength [59]. Continuous moderate-intensity training was found to have significant effects on upper body, but not lower body muscular endurance as well [59]. Furthermore, these data suggest that performing a combination of aerobic and resistance exercise during chemotherapy may result in continued participation in resistance exercise during follow-up, better patient-reported outcomes at 6 months follow-up, and better muscular strength and endurance outcomes at 12-months follow-up. Overall, the author concludes that the findings of the investigation suggests that combined exercise during and after chemotherapy may be optimal for the maintenance of the physical activity levels of breast cancer patients [59].

Improvements in the physical activity levels seen in BC survivors is important as an increase in physical activity may lead to an increase in cardiovascular fitness and a corresponding decrease in the patient’s CVD risk profile [61]. Therefore, determining if combination exercise prescriptions have any varying effects on breast cancer outcomes (e.g., recurrence, second cancers, comorbidities and overall survival) is an important direction for future research. To begin on the aforementioned path of scientific inquiry, a recent 2021 publication by Postigo-Martin and colleagues shares their plans and rationale for the Tailored Therapeutic Exercise and Recovery Strategies (ATOPE) program intervention protocol. The ATOPE intervention will look to uncover the most effective therapeutic prescription of AE (i.e., moderate continuous, high-intensity interval, etc.) for women with BC. Furthermore, the ATOPE study will compare exercise prescribed both before, and during treatment, and attempt to better elucidate the most effective strategy to attenuate chemotherapy treatment-related cardiac dysfunction [62].

In conclusion, it is clear that AT in a BC population is more widely studied than RT. Nonetheless, there is convincing evidence demonstrating the potential utility of RT when developing optimal adjuvant and neoadjuvant therapeutic exercise strategies for BC survivors. Additionally, AT has been shown to be efficacious in mitigating AC-induced cardiotoxicity when prescribed independently [50,51,52,53] or in combination with RT [58,60]. Lastly, high intensity exercise should be considered as a safe, feasible and viable option for exercise prescription; however, research aimed at uncovering evidence-based guidelines for exercise prescription in cardio-oncology patients are still needed.

**Table 4:** Combination exercise clinical trials in breast cancer survivors.

Title of the Research	Population	Exercise Regimen	Major Findings	Reference
Effects of home-based exercise training on VO_2_ in breast cancer patients under adjuvant or neoadjuvant chemotherapy (SAPS): a randomized controlled trial	BCP (*n* = 44; control: *n* = 22)	Individualized home-based combined (strength and aerobic) exercise program performed on at least 3 days per week over 27-weeks. Aerobic: 5-min warm-up, 20–40 min AE (i.e., walking/stationary bike) and a 5-min cool-down Resistance: performed 1 time each week consisting of exercise targeting 5 core muscle groups (i.e., abdominal, hamstring, quadriceps, triceps and gluteus maximus)	A supervised home-based exercise strategy during chemotherapy and radiotherapy treatment may be safe, feasible and offer increases in VO_2peak_.	Cornette et al. (2016) [60]
Highly Favorable physiological responses to concurrent resistance and high-intensity interval training during chemotherapy: the OptiTrain breast cancer trial	BCP (*n* = 240; RT-HIIT: *n* = 74; AT-HIIT: *n* = 72; control: *n* = 60)	RT-HIIT group completed both resistance and high-intensity interval exercise during each session. Participants performed 2–3 sets of 8–12 repetitions at an intensity of 80% of the patients’ estimated 1-repetition maximum. The AT-HIIT group commenced with 20 min of moderate-intensity continuous aerobic exercise at a rating of perceived exertion (RPE) of 13–15 on the Borg scale. Both RT-HIIT and AT-HIIT concluded with 3 × 3 min bouts of HIIT at an RPE of 16–18 interspersed with ~1 min of recovery.	Sixteen weeks of RT-HIIT significantly improved muscle strength and reduced pain sensitivity. Both exercise programs (RT-HIIT & AT-HIIT) were well tolerated and equally efficient in preventing increases in body mass and declines in cardiorespiratory fitness	Mijwel et al. (2018) [58]
Effects of exercise dose and type during breast cancer chemotherapy on longer-term patient-reported outcomes and health-related fitness: A randomized controlled trial	*n* = 301 BCP split between 3 intervention groups (STAN: *n* = 96; HIGH: *n* = 101; and COMB: *n* = 104)	STAN: participants that complete at least 60 min of aerobic exercise weekly. HIGH: Participants that complete at least 120 min of aerobic exercise weekly. COMB: Participants that complete at least 60 min of aerobic exercise weekly AND complete at least 2 sessions of prescribed resistance training weekly	COMB was significantly superior to: STAN, for sleep quality at 6-month follow-up (*p* = 0.027); HIGH, for upper body muscular endurance at 12-month follow-up (*p* = 0.020); HIGH, for meeting the resistance training guideline at 6-month follow-up (*p* = 0.006). Meeting the combined exercise guideline during follow-up was significantly associated with better patient-reported outcomes and health-related fitness.	An et al. (2019) [59]
Attenuating Treatment-Related Cardiotoxicity in Women Recently Diagnosed with Breast Cancer via a Tailored Therapeutic Exercise Program: Protocol of the ATOPE Trial	BCP (*n* = 120; exercise: *n* = 60)	Exercise program will consist of a 12- to 18 session-program lasting 6–8 weeks, depending on the treatment scheduled for each patient, supervised (1-on-1) program of therapeutic exercise that consists of multimodal therapeutic exercise (aerobic, strength, motor control exercises, myofascial techniques, and breathing exercises) implemented.	The ATOPE program will demonstrate that exercise intervention initiated at the time of diagnosis is needed when preventing or mitigating cardiotoxicity in women with breast cancer.	Postigo-Martin et al. (2021) [62]

BCP = breast cancer population; AE = aerobic exercise; VO_2peak_ = peak oxygen uptake; RT = resistance training; HIIT = high intensity interval training

## 8. Current Guidelines and Goals of Exercise in Breast Cancer Survivors

The most recent exercise guidelines for cancer patients from the ACSM International Multidisciplinary Roundtable recommends 3 moderate-intensity aerobic training sessions a week with a minimum duration of 30 min each [27]. In addition, it is recommended to complete strength training twice or thrice weekly that targets all major muscle groups [27]. For these strength training sessions, it is recommended to start an exercise program with 16 sessions, starting at low resistance. It is recommended the resistance is progressively built over weeks in small increments. If breaks are taken during the course of RT, it is advised to return to the resistance used two weeks prior for every week of break taken. For example, if a 3-week break is taken during RT, it is recommended the breast cancer survivor return to the weight used 6-weeks ago for their RT. In the case, cancer survivors are unable to meet the aerobic and resistance exercise targets, the United States Department of Health and Human Services (US DHHS) Physical Activity Guidelines for Americans recommends as much physical activity as their abilities and conditions allow [63]. US DHHS emphasizes the idea that some physical activity is better than none and the goal for those unable to fulfill the above guidelines due to chronic illnesses is to limit inactivity as much as possible [63].

Further, the US DHHS Physical Activity Guidelines compiled the literature on benefits of exercise in breast cancer survivors [63]. The report showed evidence indicating that physical activity in BC survivors is associated with lower risks of both BC associated mortality as well as all-cause mortality. Additionally, this benefit was seen in a dose-dependent manner meaning with increasing amounts of physical activity, there was a further decrease in mortality within this patient population. Further, subgroup analyses revealed reductions in all-cause mortality due to physical activity were present in breast cancer survivors who were normal weight, overweight, or post-menopausal. It also showed reduction in BC associated mortality due to physical activity in BC survivors who were pre-menopausal, post-menopausal, and overweight or obese [63].

These US DHHS Physical Activity Guidelines were created to meet the ACSM-directed objectives of exercise prescription in cancer survivors. These objectives are listed in Table 5. Of note, objectives 1, 4, 5, and 8 have relevance to reducing the cardiovascular risk and improving cardiorespiratory fitness in breast cancer patients receiving cardiotoxic therapies, such as anthracyclines.

## 9. Contraindications and Considerations of Exercise Programs in Cancer Patients

While the ACSM Guidelines support the safety of exercise programs during and after cancer therapy in women with BC, some considerations are recommended to enhance safety in certain groups. Firstly, women who have had BC treatment that led to issues with arm and shoulder areas are recommended to receive care for those concerns prior to engaging in upper body training. If swelling and/or other symptoms in the arm/shoulder area develop during the course of exercise, upper body exercises should be limited until these issues are resolved through consultation with professionals and the implementation of appropriate medical treatment. Secondly, for breast cancer patients with lymphedema, it is recommended a compression garment is worn during exercise to reduce the risk of injury. Thirdly, BC survivor’s post-surgery should be provided an adequate time to heal prior to engaging in exercise, which can be as long as 8 weeks. Fourthly, individuals with known cardiac conditions, whether primary or secondary to cancer treatment, should receive a medical assessment on safety of engaging in exercise. It is also recommended they have additional supervision for safety. Further, evaluation of fracture risk should be assessed prior to engaging in exercise for BC survivors on hormonal therapy, those with bone metastases, and any survivors with a diagnosis of osteoporosis. Finally, it is recommended all cancer survivors, especially those considered immunocompromised, take the appropriate infection prevention measures when exercising at public gyms to prevent the spread of infections. If at any point, cancer survivors are suffering from extreme forms of anemia, ataxia, and/or fatigue, exercise is not recommended.

## 10. Calls to Action

While many cardiac rehabilitation programs exist to improve outcomes for heart disease patients, these do not meet the specific needs of cancer patients. As cancer patients are at an elevated risk of cardiotoxicity they represent a unique patient population, thus it is necessary for exercise programs reflect their individual medical needs (e.g., cancer stage, treatment regime and status) and psychosocial needs. In America, there are LiveStrong programs which are a resource provided by community-based YMCAs for cancer patients. These programs are staffed by ACSM-certified trainers who are formally trained in working with cancer patients with a variety of different diagnoses, stages of cancer, and therapies. While Canada does have a similar program called Wellspring, which offers programs at no charge for patients at any stage of cancer, its centers are limited to two of the provinces (Alberta and Ontario). Individual provinces tend to have their own exercise programs for cancer survivors, but they can vary widely from province-to-province. As such, there is still an immediate need for a comprehensive and widely accessible program for women with breast cancer in all parts of Canada.

With the current COVID-19 pandemic, the challenges of implementing and accessing exercise programs have compounded. In this setting, multiple studies have investigated the benefits of virtually supported home-based exercise programs within the cancer population [60,65,66,67,68]. Within Canada, the Exercise for Cancer to Enhance Living Well (EXCEL) and Activating Cancer Communities through an Exercise Strategy (ACCESS) research projects have been aiming to create an accessible and effective virtually-delivered exercise program for all cancer survivors within the country. 

Most importantly, the Exercise Guideline for Cancer Survivors Consensus Statement from International Multidisciplinary Roundtable (2019), calls for the need for more research on exercise prescription on cardiac function in cancer patients [25]. The guidelines were able to show with strong evidence that exercise can benefit anxiety, depressive symptoms, fatigue, health-related quality of life, lymphedema, and physical health. However, there was insufficient evidence to show exercise was beneficial in cardiotoxicity and labelled this as a gap in knowledge for the research community to fill. To this extent, there are several randomized control clinical trials currently underway both in Canada and internationally that are investigating the benefits of exercise in breast cancer patients (ClinicalTrials.gov Identifier: NCT02796365, NCT03964142, NCT093850171, NCT03748550, NCT02842658).

## 11. Conclusions

To summarize, this review article provides a comprehensive overview of the current literature relating to the role of exercise in improving cardiovascular outcomes in the setting of anthracycline-mediated cardiotoxicity. Pre-clinical studies have shown aerobic and/or resistance exercises exert their cardioprotective benefits in the setting of anthracycline treatment primarily by reducing oxidative stress and apoptosis. Clinical trials within this setting have shown aerobic and resistance exercises are safe and can improve cardiorespiratory fitness and systolic function. However, as some studies have presented contrasting evidence, further research is warranted for optimized exercise prescription taking into consideration the frequency, intensity, time, and type of exercises. While exercise guidelines exist for women with BC undergoing chemotherapy, its implementation remains an area for improvement. 

## Figures and Tables

**Table 3:** Aerobic Exercise Clinical Trials in Breast Cancer Survivors.

Title of the Research	Population	Exercise Regimen	Major Findings	Reference
Exercise training improves cardiopulmonary and endothelial function in women with breast cancer: findings from the Diana-5 dietary intervention study	BCP (*n* = 51; control: *n* = 26)	Moderate endurance exercise (60–70% VO_2peak_) 3x/wk for 3 months	In BCP, endurance exercise training improves endothelial function and cardio-pulmonary functional capacity at 12-month follow-up	Giallauria et al. (2016) [44]
Proactive effects of acute exercise prior to doxorubicin on cardiac function of breast cancer patients: A proof-of-concept RCT	BCP (*n* = 24; control: *n* = 11)	Single-bout of vigorous intensity (70% HRR) for 30 min (with 10-min warm-up	Initial dose of doxorubicin treatment is associated with acutely increased NT-proBNP and decreased systolic function. An exercise session performed 24 h prior to treatment attenuated NT-proBNP release and increased systolic function	Kirkham et al. (2017) [49]
The effect of an aerobic exercise bout 24 h prior to each doxorubicin treatment for breast cancer on markers of cardiotoxicity and treatment symptoms: an RCT	BCP (*n* = 24; control: *n* = 11)	Single-bout of vigorous intensity (70% HRR) for 30 min (with 10-min warm-up performed prior to each of 4 treatment doses of doxorubicin	An exercise bout performed 24 h prior to each doxorubicin treatment did not have an effect on markers of subclinical cardiotoxicity, but had a positive systemic effect on hemodynamics, musculoskeletal symptoms, mood, and body weight.	Kirkham et al. (2018) [50]
Exercise to prevent anthracycline-based cardiotoxicity (EXACT) in individuals with breast or hematological cancers: a feasibility study protocol	BCP (*n* = 20; no control group)	Low-moderate intensity exercise (35–55% HRR) for 20–45 min performed on 2 (non-consecutive) days per week over 12 weeks	An individualized aerobic exercise strategy is a safe, effective and feasible program that can be effectively used to explore the effects of exercise on chemotherapy-induced cardiotoxicity	Keats et al. (2016) [51]
Feasibility of high intensity interval training in patients with breast cancer undergoing anthracycline chemotherapy: a randomized control trial	BCP (*n* = 30; control: *n* = 15)	HIIT (90%/10% PPO) performed 3x/week over 8-weeks (24 sessions)	HIIT is a feasible exercise intervention to maintain VO_2peak_ in breast cancer patients receiving anthracycline-based chemotherapy	Lee et al. (2019) [52]
Exercise intensity and cardiovascular health outcomes after 12 months of football fitness training in women treated for stage I-III breast cancer: Results from the football fitness After Breast Cancer (ABC) randomized controlled trial	BCP (*n* = 68; control: *n* = 22)	Football Fitness sessions comprised a warm-up, drills and 3–4 × 7 min of small-sided games. Exercise intensity ranged between four fitness zones (60–70%, 70–80%, 80–90%, and >90% HRmax) with the greatest amount of time dedicated to the highest intensity zone (>90% HRmax) during a given exercise session	Self-perceived health-related limitations on daily activities were improved after 6 months. 1 year of Football Fitness training comprising of 1 weekly training session on average did not improve cardiorespiratory fitness, blood pressure, blood lipids, fat mass, or HRrest.	Uth et al. 2020 [53]

BCP = breast cancer population; VO_2peak_ = peak oxygen uptake; HRR = heart rate reserve; NT-proBNP = *n*-terminal pro-brain natriuretic peptide; HIIT = high intensity interval training; PPO = peak power output; HRmax = maximum heart rate; HRrest = resting heart rate.

**Table 5:** Objectives of Exercise Prescription in Breast Cancer Survivors. Adapted from Schmitz et al. [64].

1. Restoring and improving strength, physical function, aerobic capacity, and flexibility
2. Improving body image and quality of life
3. Improving body composition
4. Improving muscular, cardiorespiratory, cognitive, neurological, endocrine, and psychosocial functions
5. Improving the ability to cope with the physical and psychological impacts of current and future anti-cancer therapies
6. Improving the ability to cope with the chronic anxiety associated with recurrence of cancer
7. Reducing likelihood of cancer recurrence and/or development of other cancers
8. Preventing long-term and late effects of anti-cancer therapies
